# Cases

**Published:** 1882-07

**Authors:** E. Rushmore

**Affiliations:** Plainfield, N. J.


### CLINICAL BUREAU.

### CLINICAL CASES.

E. Rushmore, M. D., Plainfield, N. J.

Case I. In the spring of 1881 I suffered for several days from pain in the second bicuspid in the left upper jaw. Chamomilla relieved for twenty-four hours and then had no effect; several other remedies did no good. The pain was very bad at night, preventing sleep. I noticed that on getting out of bed the pain became less; hoping this symptom would prove a clue to the remedy, I went down-stairs to my office to hunt it up. Opening my repertory I found under toothache, better from getting out of bed: Oleand., Sabin; turning to the proving of Oleander, I read: "Constant toothache during the night * * * in the first left molar and at times in the next hollow tooth; this toothache immediately ceased on rising from bed and immediately reappeared as soon as he returned to bed, with anxiety as if he were to die, with frequent micturition, qualmishness and heat of the left cheek." Anxiety and qualmishness I had not, but frequent micturition and heat of the left cheek were marked features of the case. I put a few globules of Oleander 200 (Durham), on the tongue, went back to bed and fell asleep in a few minutes. On waking in the morning there was no pain and it has never returned.

Case II. A boy had toothache, the only reported peculiarity being worse from sweet things, "like molasses." My repertory gave Nat. carb., Sepia; of these Hering mentions only Nat. carb. as having that characteristic. I therefore thought it probably more strongly characteristic of Nat. carb., and sent him a high potency of the same (900th of Fincke or higher). The report next day was that the medicine stopped the pain at once and that it had not returned.

Case III. A lady had for several days diarrhœa after dinner and supper; before the stool, colicky pains; during stool, urging and tenesmus; after stool, burning in the anus. From a case reported by Dr. C. Lippe in *The Organon*, I had noted in my repertory: "Diarrhœa, worse after dinner and supper, not after breakfast: Thrombidium." My patient had no disturbance after breakfast, but the stool would be repeated several times after dinner and supper. The concomitants all being found under Thrombidium, I gave her one dose of a few globules of Thrombidium, $71^{m}$ (Fincke), in the evening. All the trouble disappeared the next day.

CASE IV. September 22d, 1881. Mr. M. has had for six months a dull, heavy sensation in the region of kidneys, worse in the morning in bed, relieved by wrapping up. The pain lasts all day, sometimes worse while walking or in rising from a seat; urine light colored. Headache almost daily with obscured sight, and sense of emptiness in the head. Has vertigo, often coming suddenly, while walking, felt from the forehead to vertex, with falling forwards. Nux vom., $94^{m}$ (Fincke), one dose, dry, in the evening.

September 29th. Reports headache and vertigo all gone and the back a great deal better; does not use extra covering at night, as before, and sleeps till it is time to get up. No medicine. After that he reported himself well.

